# Development and Performance Analysis of a U-Shaped Fiber Optic Sensor for Soil Water Content Measurement

**DOI:** 10.3390/s26175619

**Published:** 2026-09-04

**Authors:** Alireza Sabetimani, Rashid Bashir, Reza Rizvi

**Affiliations:** Lassonde School of Engineering, York University, Toronto, ON M3J 1P3, Canada; alisabet@yorku.ca (A.S.); reza.rizvi@lassonde.yorku.ca (R.R.)

**Keywords:** U-shaped fiber optic sensor, soil water content, gravimetric water content, optical sensing, photodiode output, wavelength response, soil moisture calibration

## Abstract

Accurate soil water content measurement is essential for irrigation management, soil water retention studies, and geotechnical monitoring. This study developed and evaluated a U-shaped silica fiber optic sensor for soil water content measurement using optical transmission responses at 500, 940, and 1450 nm wavelengths. The sensor was fabricated using a bent optical fiber coupled with LED light sources and a photodiode-based detection system. Laboratory tests were conducted on three soil samples under different gravimetric water content (GWC) levels, and the photodiode outputs were normalized to compare sensor behavior across wavelengths and soil types. Under each condition, five consecutive readings were obtained at three probe locations. The five readings were treated as technical repeats and first averaged at the location level, after which the three location responses were averaged to obtain one representative response for each soil water content condition. Linear, quadratic, cubic, and exponential forward models were compared, and the cubic model provided the strongest descriptive fit for the combined-soil dataset, particularly at 1450 nm ($R^{2}=0.8956$, adjusted $R^{2}=0.8799$, RMSE = 0.0717). For practical water-content estimation, separate direct inverse linear models were developed and evaluated using grouped leave-one-water-content-level-out cross-validation. The corresponding RMSE values were 3.41, 3.37, and 3.20 percentage points of GWC at 500, 940, and 1450 nm, respectively. The 1450 nm wavelength also showed the highest range-dependent sensitivity. Time-dependent measurements also showed repeatable response patterns during drying, demonstrating the sensor’s ability to track water content redistribution. Overall, the developed U-shaped fiber optic sensor showed a measurable and repeatable response to soil water content variation and has potential as a simple optical sensing approach for soil water content monitoring.

## 1. Introduction

In geotechnical engineering, accurately measuring soil water content is crucial because water content in the unsaturated zone directly controls matric suction and effective stress, which govern soil shear strength and the stability of slopes, embankments, and foundations [1]. Even modest changes in moisture can alter performance; for example, rainfall infiltration can change matric suction and thus shear strength, changing the risk of slope failures, whereas excessive drying can induce shrinkage and desiccation cracking that compromise soil integrity [2]. Accordingly, continuous monitoring of volumetric water content is essential for managing these moisture-driven changes and ensuring the safety and performance of geotechnical systems under variable environmental conditions [3,4]. Conventional moisture measurement methods have significant limitations in the field. The standard gravimetric oven-drying test, while highly precise, is destructive and labor-intensive. Because it requires physical soil extraction, it is impractical for continuous in situ monitoring [5]. Furthermore, this method provides only a static, single-point snapshot, failing to capture transient moisture dynamics driven by infiltration or evaporation [5]. To address these temporal gaps, a variety of in situ sensors are utilized to measure volumetric water content; however, all possess inherent limitations. Time Domain Reflectometry (TDR), for instance, typically delivers high precision and accuracy, yet its performance degrades in saline or clay-rich soils. In such environments, signal attenuation and dielectric dispersion obscure the waveform, compromising measurement reliability. Moreover, TDR systems are often cost-prohibitive, making them difficult to scale across dense field monitoring networks [6]. Capacitance-based sensors, such as Frequency Domain Reflectometry (FDR) devices, offer a more cost-effective and easily deployable alternative [7]. Nevertheless, they are highly susceptible to fluctuations in temperature, soil electrical conductivity, and bulk density, requiring rigorous site-specific calibration to prevent erroneous readings under variable conditions [7]. Similarly, while neutron probes excel at deep moisture profiling, they carry strict safety and regulatory burdens, and they lack the near-surface sensitivity crucial for assessing shallow geotechnical stability [8]. Ultimately, these compounding vulnerabilities in existing reflectometry and nuclear techniques hinder the collection of reliable, long-term moisture data in complex geotechnical environments, underscoring the critical need for more robust sensing solutions.

Fiber-optic sensors have gained increasing attention for monitoring in challenging geotechnical and environmental conditions because of their small size, high sensitivity, corrosion resistance, and immunity to electromagnetic interference [9,10]. In soil moisture monitoring, several fiber-optic approaches have been investigated, including actively heated distributed temperature sensing (AH-DTS) [11], actively heated fiber Bragg grating (AH-FBG) sensors [12], long-period grating sensors, modified fiber structures, coated or side-polished fibers, and refractive-index-based optical probes [13,14,15,16,17,18,19]. Actively heated fiber-optic methods are useful for distributed or quasi-distributed monitoring because they estimate soil water content from the thermal response of the soil surrounding the fiber [13,14,15]. However, these methods measure moisture indirectly through heat transfer behavior and generally require controlled heating, relatively high energy demand, optical interrogation equipment, and soil-specific model development. These requirements can limit their practicality for simple, low-cost, point-based monitoring systems.

Other localized fiber-optic soil moisture sensors have been developed using refractive-index variation, special coatings, long-period gratings, D-shaped fibers, and U-bend plastic optical fibers [17,18,19,20,21]. These studies demonstrate that local changes in moisture around an optical fiber can produce measurable changes in transmitted or guided light. However, many of these sensors require specialized fiber modification, thin-film coating, grating fabrication, or optical interrogation systems, which increase fabrication complexity and cost. In addition, limited work has evaluated a simple multi-wavelength LED–photodiode U-shaped silica fiber system for soil water content measurement for different soils. The U-shaped geometry is important because bending the fiber increases optical attenuation and enhances the interaction between the guided evanescent field and the surrounding soil–water medium. Therefore, changes in water content near the curved sensing region can be detected as changes in transmitted optical intensity. Table 1 compares representative soil water content measurement technologies reported in the literature.

Recent developments in optical-fiber moisture sensing have increasingly focused on modified fiber geometries and functional coatings to enhance the interaction between the guided optical field and the surrounding environment. U-shaped fibers are particularly attractive because bending increases the interaction of the evanescent field with surface coatings and external media. Hsu et al., for example, developed a U-shaped humidity sensor coated with electrospun Pd/TEA/PVA, demonstrating the potential of combining bend-induced optical sensitivity with a hygroscopic sensing layer [26]. Earlier work using coated U-shaped fibers similarly demonstrated their suitability for relative-humidity measurement. In parallel, Wang et al. incorporated a GeS sensitizing layer into a U-shaped surface plasmon resonance (SPR) sensor [27], while Ye et al. employed an ITO coating to enhance the surface electromagnetic field and refractive-index sensitivity [28]. Although these latter studies primarily targeted refractive-index sensing rather than direct moisture measurement, they demonstrate the high sensitivity achievable with U-shaped SPR configurations and their potential integration with moisture-responsive materials.

Modified optical fibers have also been investigated for direct soil-moisture sensing. Zulkapli and Adnan demonstrated that the optical-fiber response varies with soil type and sensor geometry, highlighting the importance of soil-specific calibration [29]. Tien et al. developed a SnO_2_-coated D-shaped fiber based on lossy-mode resonance and successfully evaluated it in soil, demonstrating high sensitivity to changes in the surrounding moisture condition [20]. More recently, Sun et al. combined a hygroscopic calcium-alginate coating with a D-shaped fiber and multipeak wavelength-shift averaging to improve humidity-measurement accuracy [30]. Collectively, these studies indicate that sensing performance can be enhanced through the combined optimization of fiber geometry, functional materials, and optical interrogation methods. Nevertheless, relatively limited research has examined the performance of simplified fiber-optic configurations for quantitative soil water content measurement under direct soil contact. In particular, the effects of soil characteristics, sensor–soil interaction, and measurement configuration remain important considerations for developing robust and transferable fiber-optic soil-moisture sensors.

Beyond these application-oriented developments, recent studies have also focused on the fabrication, modelling, and reproducibility of U-shaped optical-fiber sensors. Lang et al. investigated a low-cost fabrication method and demonstrated that the bending diameter strongly affects optical transmission and sensing stability [31]. Cong et al. developed a U-shaped tapered no-core fiber humidity sensor with an MXene coating, demonstrating the potential of U-shaped structures for enhanced moisture-sensitive interaction [32]. More recently, Danny et al. developed and experimentally validated a ray-optics model describing evanescent-wave absorption in U-bent fiber sensors [33]. Hirakawa et al. also introduced an automated thermal-forming process for producing U-shaped silica probes with improved geometric and optical reproducibility [34].

These studies highlight the growing potential of optical fiber platforms for moisture-related sensing across a range of configurations and operating principles. At the same time, the performance of U-shaped fiber sensors is strongly governed by factors such as bend geometry, fiber structure, surface condition, and fabrication reproducibility. Despite these advances, most recent U-shaped systems have been developed primarily for refractive-index, humidity, biosensing, or plasmonic applications. Their use for direct soil water content estimation, particularly with a simple intensity-based LED–photodiode configuration, remains comparatively limited.

The aim of this study is to design, develop, and evaluate a simple U-shaped fiber optic sensor for soil water content measurement. The proposed sensor uses a U-shaped silica fiber probe coupled with low-cost LED light sources and a photodiode-based detection system, avoiding the need for active heating, fiber Bragg grating interrogation, or spectrometer-based measurement. The key idea explored in this work is whether a bent silica fiber probe, combined with selected visible and near-infrared wavelengths, can provide a measurable and repeatable optical response to soil water content variation. Specifically, this study includes: (1) the design and fabrication of a silica U-shaped fiber sensing probe to enable evaluation at 1450 nm, where water absorption is strong, unlike many low-cost U-bend optical sensing approaches based on plastic fibers or modified fiber structures; (2) integration of the probe with a multi-wavelength LED source, concave mirror coupling unit, and photodiode detection system; (3) evaluation of the normalized optical response at 500, 940, and 1450 nm to compare visible-range scattering effects, moderate near-infrared water sensitivity, and stronger near-infrared water absorption within the same sensing platform; and (4) evaluation of soil-specific and combined model performance using three sandy soils with different particle-size ranges and observed particle colors.

## 2. Materials and Methods

### 2.1. Sensor Design and Working Principle

**Figure 1:**
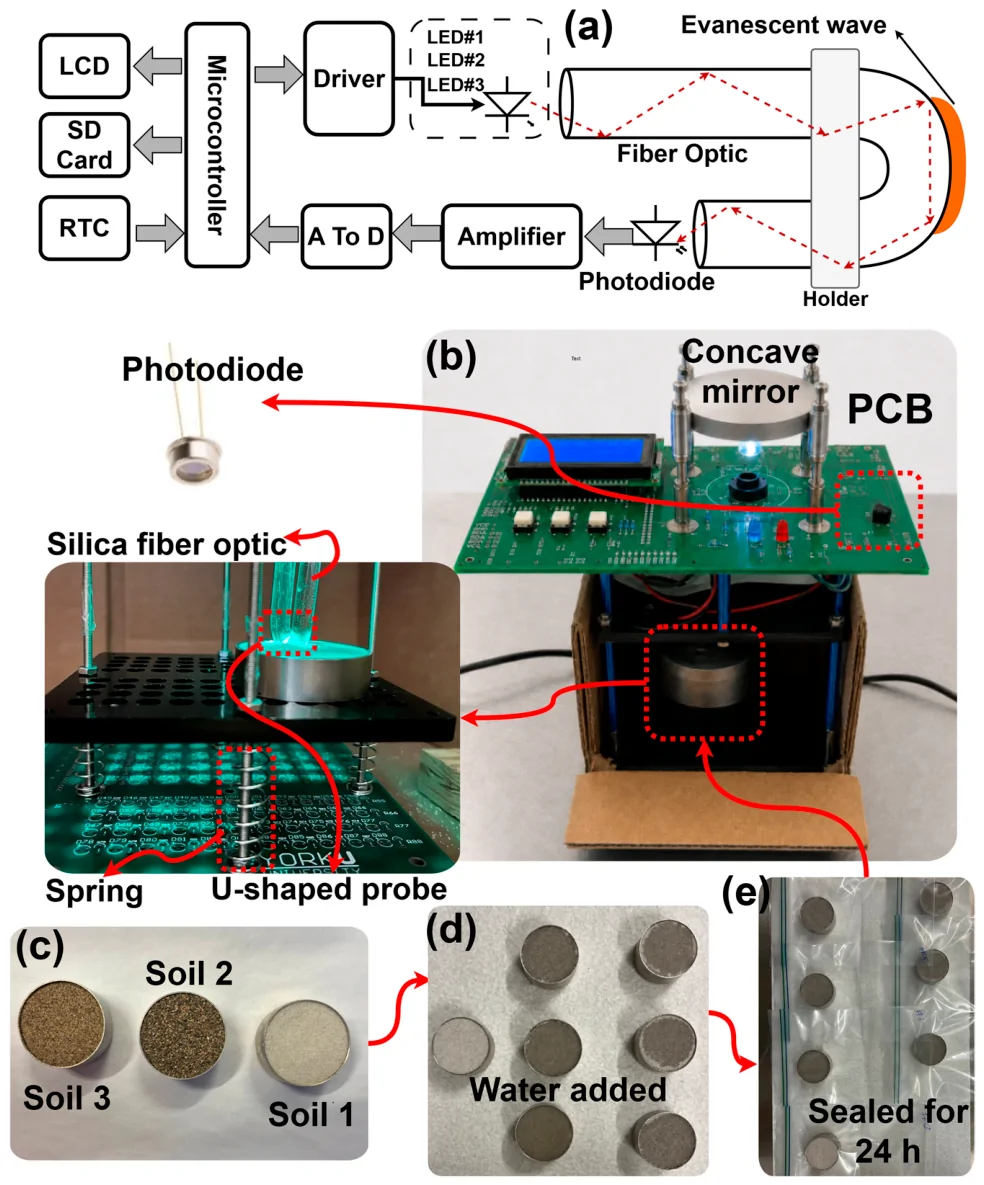
Overview of the U-shaped fiber-optic soil moisture sensor: (**a**) schematic of the sensing system and signal acquisition components; (**b**) assembled prototype showing the PCB, LCD, LEDs, concave mirror, photodiode, U-shaped silica fiber probe, and Spring-supported platform; (**c**) tested soil samples; (**d**) prepared soil specimens after water addition; and (**e**) sealed specimens stored for 24 h to allow moisture equilibration before testing.

**Figure 2:**
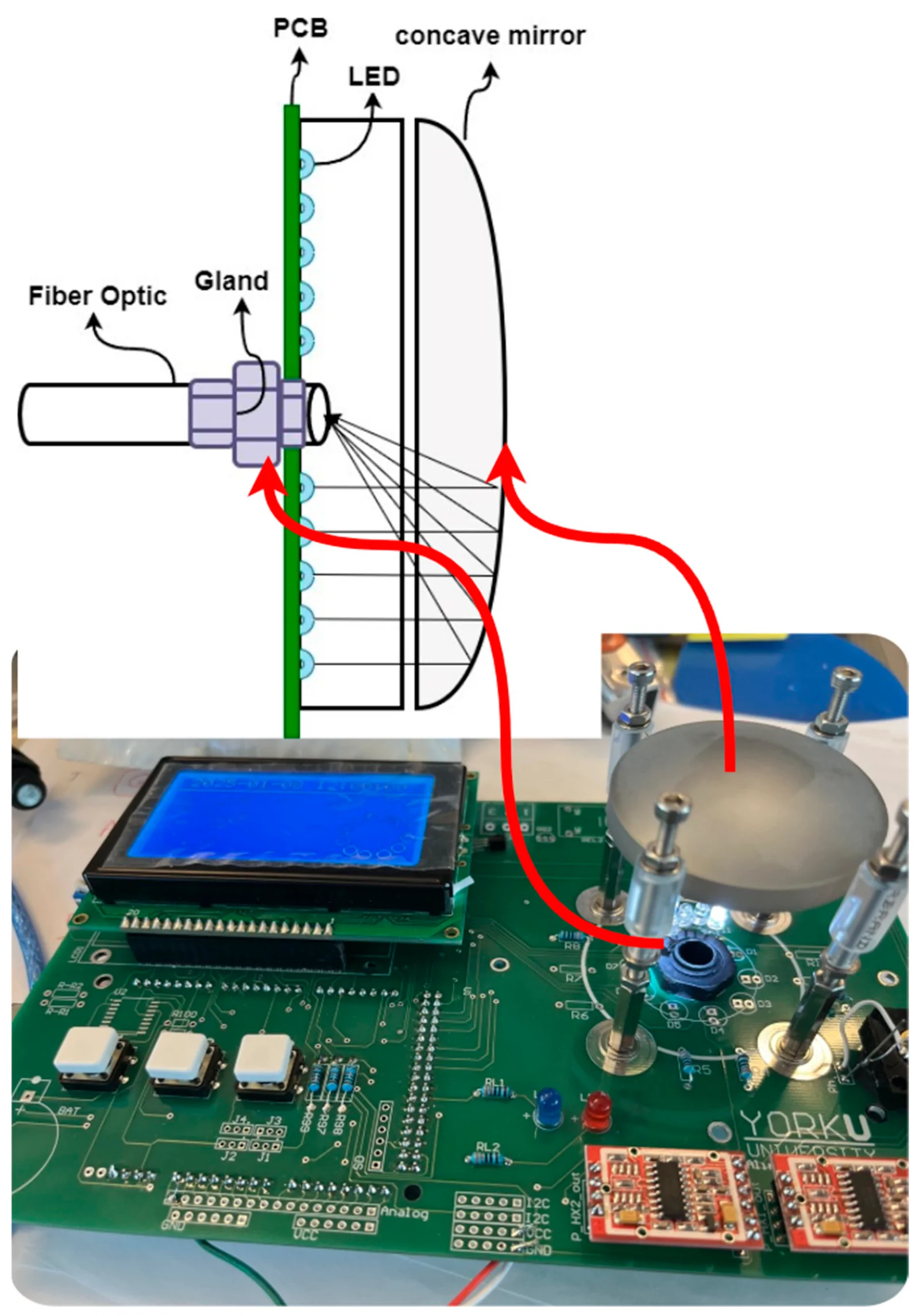
Optical coupling unit of the developed sensor, showing the PCB-mounted LEDs, concave mirror, gland connector, and aligned fiber optic entrance.

The proposed U-shaped fiber optic soil moisture sensor was designed as a fully integrated optoelectronic platform. At its core, an ATmega328 (Microchip Technology Inc., Chandler, AZ, USA) microcontroller orchestrates the system [35], interfacing with an LED driver, multiple light-emitting diodes (LEDs), whose specifications are summarized in Table 2, a photodiode, a signal amplifier, and an analog-to-digital converter (ADC). To support autonomous data collection, the platform is further equipped with a real-time clock (RTC), an SD card storage module, and an LCD interface. In operation, the microcontroller sequentially triggers the LED driver to emit distinct wavelengths. This light is coupled into the silica optical fiber and propagates toward the U-shaped sensing region embedded within the soil sample (Figure 1). To optimize optical coupling, the LEDs are mounted on a custom printed circuit board (PCB) and positioned adjacent to a concave mirror (Figure 2). A protected-aluminum concave mirror with a diameter of 50 mm and a focal length of 25 mm (Edmund Optics, Barrington, NJ, USA, Stock No. 43–469) was used to redirect and focus the LED light onto the entrance face of the 6 mm silica fiber entrance, while a dedicated gland connector secures the fiber to maintain strict alignment. Ultimately, this robust optomechanical arrangement minimizes signal noise induced by physical movement or poor light coupling, enabling the reliable evaluation of multiple wavelengths through a single sensing probe. The experiments were conducted under stable laboratory conditions at 23 °C and 10% relative humidity. Short, sequential LED activation minimized self-heating.

The working principle of the sensor is based on the change in transmitted light intensity caused by the interaction between the guided light and the surrounding soil–water medium. When light travels through the U-shaped section of the fiber, part of the optical field extends beyond the fiber surface as an evanescent wave [19,20]. Because this region is in direct contact with the soil, changes in soil water content modify the refractive index condition around the fiber surface. As the water content increases, the optical loss in the bent sensing region changes, which affects the amount of light reaching the photodiode. Bending the optical fiber into a U-shape enhances attenuation and increases the interaction between the guided light and the surrounding medium, making this structure highly effective for moisture and soil-moisture sensing [19,21]. The transmitted light was detected using a Hamamatsu G10899-03K visible-enhanced InGaAs PIN photodiode (Hamamatsu Photonics K.K., Hamamatsu, Japan) with a 3.0 mm active area and a spectral response range of 500–1700 nm, covering all three investigated wavelengths. The received signal is then amplified, converted to a digital value, displayed on the LCD, and stored on the SD card with time information from the RTC. Therefore, the measured photodiode output can be calibrated against gravimetric water content (GWC) and used as an indicator of soil water content variation in the tested soil samples, which differed in particle-size range and visual appearance.

The U-shaped sensing probe was fabricated from a coreless solid fused-silica optical rod with an overall diameter of 6.0 mm. The optical rod sensing region was uncoated and had no polymer jacket, allowing its external silica surface to interact directly with the surrounding soil–water medium. The selected bending region was gradually heated with a micro-torch until the silica became sufficiently softened for shaping. The fiber was then carefully formed into a U-shaped geometry while maintaining symmetry between the two vertical arms and a smooth curved sensing region at the bottom. After forming, the probe was allowed to cool naturally at room temperature to preserve the final geometry and reduce thermal stress. The fabricated probe had an approximate bend radius of 6 mm, estimated from the final probe geometry. The U-shaped section served as the active sensing region because bending increases optical attenuation and enhances the interaction between the guided light and the surrounding soil medium [19,21]. As soil water content changes around this curved region, the optical loss changes, resulting in a measurable variation in the photodiode output.

A spring-supported loading platform was incorporated into the experimental apparatus to control the mechanical contact between the soil specimen and the U-shaped probe. As shown in Figure 1, multiple springs under compression were distributed beneath the perforated platform. The springs allowed limited vertical displacement and reduced the application of sudden or excessive compressive force during probe embedment. This arrangement also helped maintain repeatable contact between the curved sensing region and the soil during successive measurements. Therefore, the probe was not subjected to uncontrolled manual compaction during testing.

### 2.2. Soil Samples

Three sand samples, referred to as Soil 1, Soil 2, and Soil 3, were used to evaluate the performance of the U-shaped fiber optic sensor over a range of GWC levels (Figure 1). The samples differed mainly in particle-size range and color. Soil 1 was a white Sand 30/40, Soil 2 was a dark brown Sand 40/50, and Soil 3 was a light brown Sand 100/200. These differences are critical because particle size dictates pore structure, water retention, and soil–fiber contact, while soil color influences light reflection, scattering, and absorption around the sensor. For sample preparation, a known mass of dry soil was first placed in each container, and the required amount of water was added to obtain the target gravimetric water content. To improve consistency among tests, all specimens were prepared using the same container geometry, soil mass, filling procedure, preparation time, and measurement protocol. The soil and water were thoroughly mixed to promote uniform water distribution. After mixing, the prepared samples were sealed for 24 h to allow moisture equilibration and to minimize evaporation before sensor measurements. The prepared specimens were tested independently at each GWC level to develop a relationship between the optical response of the sensor and soil water content. Using three different sand samples allowed the sensor response to be examined for individual soils and for a combined dataset representing a wider range of sands with different particle-size ranges and visual appearances.

The GWC for each specimen was calculated using the mass-based method [36]:

(1) $$w=\frac{M_{w}-M_{d}}{M_{d}}\times 100$$

where w is the GWC (%), $M_{w}$ is the wet mass of the soil, and $M_{d}$ is the dry mass of the soil. GWC was used as the reference measurement in this study because it provides a direct mass-based determination of soil water content, whereas commercial soil moisture sensors often require soil-specific correction or validation under different soil conditions [36,37].

### 2.3. Experimental Procedure

The U-shaped fiber optic probe was installed in the prepared soil sample such that the lower outer curvature of the U-shaped sensing region was placed in direct contact with the soil. For each soil and water content level, the optical response was measured at 500, 940, and 1450 nm to evaluate the wavelength-dependent sensitivity of the U-shaped fiber optic sensor. The 500 nm wavelength was selected to represent the visible range, where soil optical response can be influenced by soil color, particle reflectance, and scattering effects [38,39]. The 940 nm wavelength was selected because it is close to the water-related absorption feature around 970 nm, which has been reported as one of the moisture-sensitive bands in the near-infrared region [40]. The 1450 nm wavelength was selected because it corresponds to a strong water absorption band associated with O–H vibration overtones, making it highly relevant for detecting soil moisture variation [40,41]. Therefore, one objective of this study was to compare whether visible-range scattering effects, moderate near-infrared water sensitivity, and stronger near-infrared water absorption produce different responses when applied within the same U-shaped fiber optic sensing platform. The LEDs were activated separately to avoid overlap between wavelengths, and the corresponding photodiode voltage was recorded for each wavelength.

For each soil sample, a series of GWC levels were prepared to evaluate the response of the U-fiber optic sensor under controlled water content conditions. Three sand samples were tested: Soil 1 was classified as white Sand 30/40, Soil 2 as dark brown Sand 40/50, and Soil 3 as light brown Sand 100/200 (Figure 1). Each soil sample was prepared at eight GWC levels: 0, 3, 5, 8, 10, 15, 18, and 20%. At each water content level, measurements were collected at three separate locations, and five repeated readings were recorded at each point to evaluate the consistency of the sensor output and the effect of probe placement. At each measurement location, the sensing system was allowed to stabilize for 30 s. This stabilization period was used only to obtain a stable optical signal and was not considered a measurement of the sensor response time. Five consecutive measurement cycles were then recorded over approximately 55 s. Each cycle consisted of sequential acquisition from the three LED wavelengths (500, 940, and 1450 nm), with an interval of about 2 s between consecutive cycles. The same acquisition procedure was used for all soil samples, water-content levels, and wavelengths. Therefore, the experimental design provided 15 readings per water content level for each soil sample (Table 3).

For each prepared soil specimen, the optical response was measured sequentially at all three wavelengths: 500, 940, and 1450 nm. The LEDs were activated one at a time to avoid overlap between wavelengths, and the corresponding photodiode output was recorded separately for each wavelength. The recorded photodiode outputs were normalized and organized as a function of GWC for each wavelength and soil type. The data from all three soils were also combined to evaluate the general response of the U-fiber sensor across soils with different particle size distributions and colors.

In addition to the tests at fixed GWC levels, evaporation tests were conducted for each sand sample to evaluate the time-dependent response of the U-fiber optic sensor during soil drying. For this test, the soil sample was initially saturated to establish a wet starting condition, and the U-shaped fiber optic sensor was installed in the prepared soil. The photodiode output was then monitored continuously as water evaporated under laboratory conditions. During the evaporation test, the 1450 nm LED was used because this wavelength showed the strongest water-sensitive response in the fixed water-content experiments. The photodiode output was recorded continuously as the soil dried under laboratory conditions. Duplicate drying tests were conducted for each soil sample to examine the repeatability and temporal trend of the optical response during drying and to verify that the sensor output followed the expected drying behavior observed from the fixed water-content results.

### 2.4. Data Analysis

To compare the sensor responses under different wavelengths and soil conditions, the raw photodiode outputs were normalized using two reference conditions: air condition and water condition. Before soil testing, the U-shaped fiber optic probe was first exposed to air and then fully immersed in water. The photodiode outputs measured in these two conditions were used as the upper and lower reference limits for normalization. The air condition represented the maximum reference signal because the fiber was surrounded by air, while the water condition represented the minimum reference signal because the surrounding medium had a higher refractive index and caused greater optical loss around the U-shaped sensing region. The normalized photodiode output was calculated as:

(2) $$I_{n}=\frac{I_{s}-I_{w}}{I_{a}-I_{w}}$$

where $I_{n}$ is the normalized photodiode output, $I_{s}$ is the photodiode output measured in the soil sample, $I_{a}$ is the reference photodiode output measured in air, and $I_{w}$ is the reference photodiode output measured in water. Therefore, the normalized value represents the relative optical response of the sensor between dry-air and fully-water reference conditions. This normalization reduced the effect of differences in LED intensity, photodiode sensitivity, and optical coupling among different wavelengths, allowing the sensor response at 500, 940, and 1450 nm to be compared more consistently. Five consecutive readings collected at each measurement location were treated as technical repeats and averaged to obtain one location-level response. The three location-level means were then averaged to obtain one representative value for each soil water content condition. Regression was therefore performed using 8 condition-level observations per soil and 24 observations for the combined dataset, with the standard deviation among the three locations representing spatial and installation variability.

The statistical analysis was first performed by considering the normalized optical response of the U-fiber sensor as the dependent variable and the GWC as the independent reference variable. This approach was selected because the sensor response is physically controlled by changes in soil moisture. Linear, quadratic, cubic, and exponential forward models were compared using $R^{2}$, adjusted $R^{2}$, RMSE, and AICc. The detailed candidate-model comparison is provided in Supplementary Table S1. Based on this comparison, a third-order polynomial regression (Equation (3)) was used to describe the sensor response. Separate soil-specific models were developed for each soil sample at each wavelength. In addition, combined soil models were generated using the data from all soils to evaluate the general applicability of the sensor. The model can be expressed as:

(3) $$Y=a_{0}+a_{1}X+a_{2}X^{2}+a_{3}X^{3}$$

where Y is the normalized optical response of the U-shaped fiber optic sensor, X is the gravimetric water content, and $a_{0}$, $a_{1}$, $a_{2}$, and $a_{3}$ are regression coefficients. The combined “all soils” analysis was used to assess whether a generalized response model could be developed across the tested soil samples, while the individual-soil models were used to evaluate soil-dependent behavior. The corresponding regression coefficients, and statistical performance indicators for each wavelength and soil sample are summarized in Tables S2 and S3.

The performance of the fitted models was evaluated using the coefficient of determination $\left(R^{2}\right)$, correlation coefficient $\left(R\right)$, residual sum of squares $\left(RSS\right)$, and *p*-value. These parameters were used to assess the agreement between the measured normalized photodiode output and the values predicted by the fitted model.

The residual sum of squares $\left(RSS\right)$ was calculated as:

(4) $$RSS=\sum_{i=1}^{n}(y_{i}-{\hat{y}}_{i}{)}^{2}$$

where $y_{i}$ is the measured normalized photodiode output, ${\hat{y}}_{i}$ is the predicted value from the fitted model, and n is the number of data points. A smaller RSS indicates a lower difference between measured and predicted values.

The coefficient of determination $\left(R^{2}\right)$ was calculated as:

(5) $$R^{2}=1-\frac{RSS}{TSS}$$

where TSS is the total sum of squares:

(6) $$TSS=\sum_{i=1}^{n}(y_{i}-\bar{y}{)}^{2}$$

and $\bar{y}$ is the mean of the measured values. The $R^{2}$ value represents the proportion of variation in the measured sensor output explained by the fitted model. A value closer to 1 indicates better model performance.

The interval sensitivity was calculated as the absolute change in normalized photodiode output divided by the corresponding change in gravimetric water content:

(7) $$S=\frac{|Y_{2}-Y_{1}|}{|w_{2}-w_{1}|}$$

where S is the interval sensitivity, $Y_{1}$ and $Y_{2}$ are the predicted normalized photodiode outputs at the limits of each interval, and $w_{1}$ and $w_{2}$ are the corresponding gravimetric water contents. Sensitivity was reported in normalized-output units per percentage point of gravimetric water content.

For practical estimation of unknown gravimetric water content, a separate direct inverse calibration was developed with GWC as the dependent variable and normalized photodiode output as the predictor. This inverse model was treated separately from the cubic forward model, which was retained only for characterization of the sensor response. Linear inverse regression was selected because it provides a unique water-content estimate and showed the most stable overall performance during grouped cross-validation. The inverse calibration was expressed as:

(8) $$W=a+bI_{n}$$

where W is the predicted GWC (%), $I_{n}$ is the normalized photodiode output, and a and b are the fitted intercept and slope, respectively. Predictive performance was evaluated using leave-one-water-content-level-out cross-validation, with RMSE and MAE reported in percentage points of gravimetric water content. Lower RMSE and MAE values indicate smaller prediction errors.

## 3. Results

**Figure 3:**
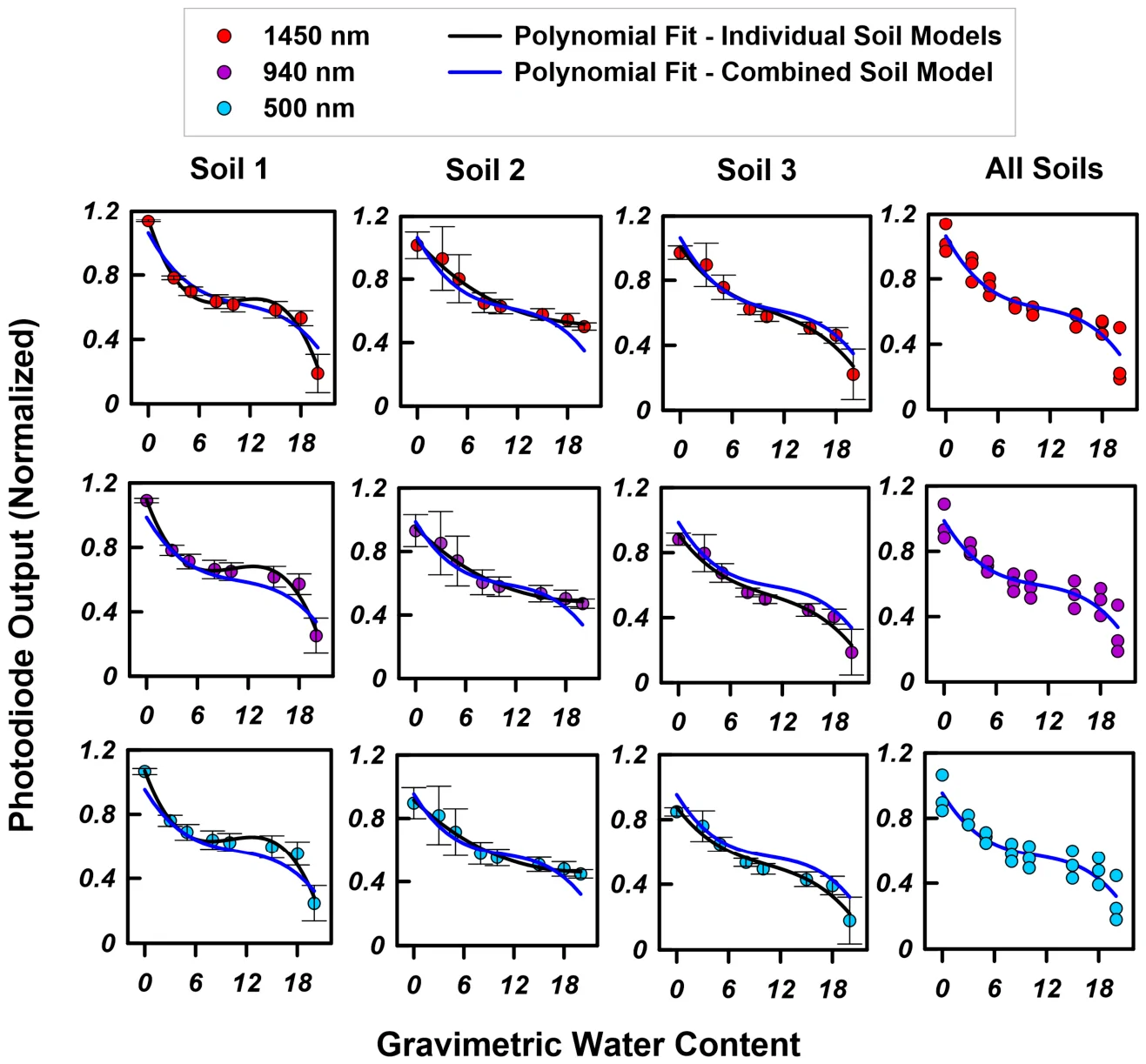
Relationship between normalized photodiode output and gravimetric water content for individual soils and the combined soil dataset at 500, 940, and 1450 nm. Rows correspond to the investigated wavelengths, while columns represent the individual soils and all soils combined. Colored markers show the experimental measurements, black curves represent the soil-specific polynomial fits, and blue curves indicate the combined-soil polynomial model.

Figure 3 shows the relationship between GWC and normalized photodiode output for the three tested soils at 500, 940, and 1450 nm. Overall, the sensor response showed a clear decreasing trend as water content increased. At low water content, the normalized photodiode output was generally close to 1, while at higher water content it decreased to lower values, commonly around 0–0.5 depending on wavelength and soil type. This indicates that increasing soil moisture changed the optical condition around the U-shaped fiber and increased the optical loss in the sensing region.

Following hierarchical averaging, regression analysis was performed using eight condition-level observations per soil and 24 observations for the combined-soil dataset. Linear, quadratic, cubic, and exponential forward models were compared, and the third-order polynomial provided the strongest overall descriptive fit. For the combined dataset, the cubic models produced $R^{2}$ values of 0.8631, 0.8649, and 0.8956 at 500, 940, and 1450 nm, respectively, with corresponding adjusted $R^{2}$ values of 0.8426, 0.8446, and 0.8799. The RMSE values were 0.0729, 0.0745, and 0.0717, respectively (Table S1). Thus, 1450 nm provided the strongest descriptive agreement with the combined condition-level data.

The combined “all soils” plots also showed a consistent negative relationship between water content and normalized photodiode output for all three wavelengths. However, more scatter was observed in the combined datasets compared with the individual soil plots. This scatter is expected because soil color, particle size, packing condition, and soil–fiber contact can affect light interaction around the fiber surface. The repeated measurements and reinstallations also produced small variations at each water content level, showing the importance of controlling probe placement during testing.

Among the tested wavelengths, 500, 940, and 1450 nm all responded to water content variation, but their response patterns were different. The near-infrared wavelengths, especially 940 and 1450 nm, showed strong moisture-dependent trends, while the visible wavelength also showed a measurable response. These results confirm that the developed U-shaped fiber optic sensor can detect soil water content changes, but wavelength-specific and soil-specific model development is needed to improve prediction accuracy. Overall, the results demonstrate the potential of the proposed sensor as a simple optical method for monitoring soil moisture under laboratory conditions.

The soil-specific cubic models provided strong descriptive fits between normalized photodiode output and gravimetric water content. The $R^{2}$ values ranged from approximately 0.958 to 0.981 across the three soils and wavelengths. Soil 1 produced the highest $R^{2}$ values, ranging from 0.978 to 0.981, while the corresponding values for Soil 2 ranged from 0.976 to 0.979 and those for Soil 3 ranged from 0.958 to 0.964 (Table S3). These results indicate that the magnitude and shape of the optical response were soil dependent, despite the general decrease in normalized output with increasing water content.

For the combined-soil dataset, the cubic models produced $R^{2}$ values of 0.8631, 0.8649, and 0.8956 at 500, 940, and 1450 nm, respectively, with corresponding adjusted $R^{2}$ values of 0.8426, 0.8446, and 0.8799. The RMSE values were 0.0729, 0.0745, and 0.0717, respectively (Table S2). Thus, the 1450 nm model provided the strongest overall descriptive agreement with the combined condition-level observations. As shown in Figure 3, the combined-soil models exhibited greater scatter than the soil-specific models, indicating that differences among the tested soils contributed to variability in the optical response.

The fitted cubic relationships were also examined for monotonicity over the experimental range of 0–20% GWC. The Soil 2, Soil 3, and combined-soil models remained monotonically decreasing, whereas the Soil 1 cubic models showed local changes in slope within the calibrated range. Consequently, the cubic models were retained primarily to characterize the nonlinear sensor response rather than for direct inversion to estimate unknown water content.

Overall, Figure 3 demonstrates that all three wavelengths responded systematically to changes in gravimetric water content, with 1450 nm providing the strongest generalized descriptive performance. The differences between the soil-specific and combined-soil relationships further indicate that soil characteristics influence the optical response and should be considered when developing generalized calibration models. Practical prediction of unknown water content was therefore evaluated separately using the direct inverse calibration.

**Figure 4:**
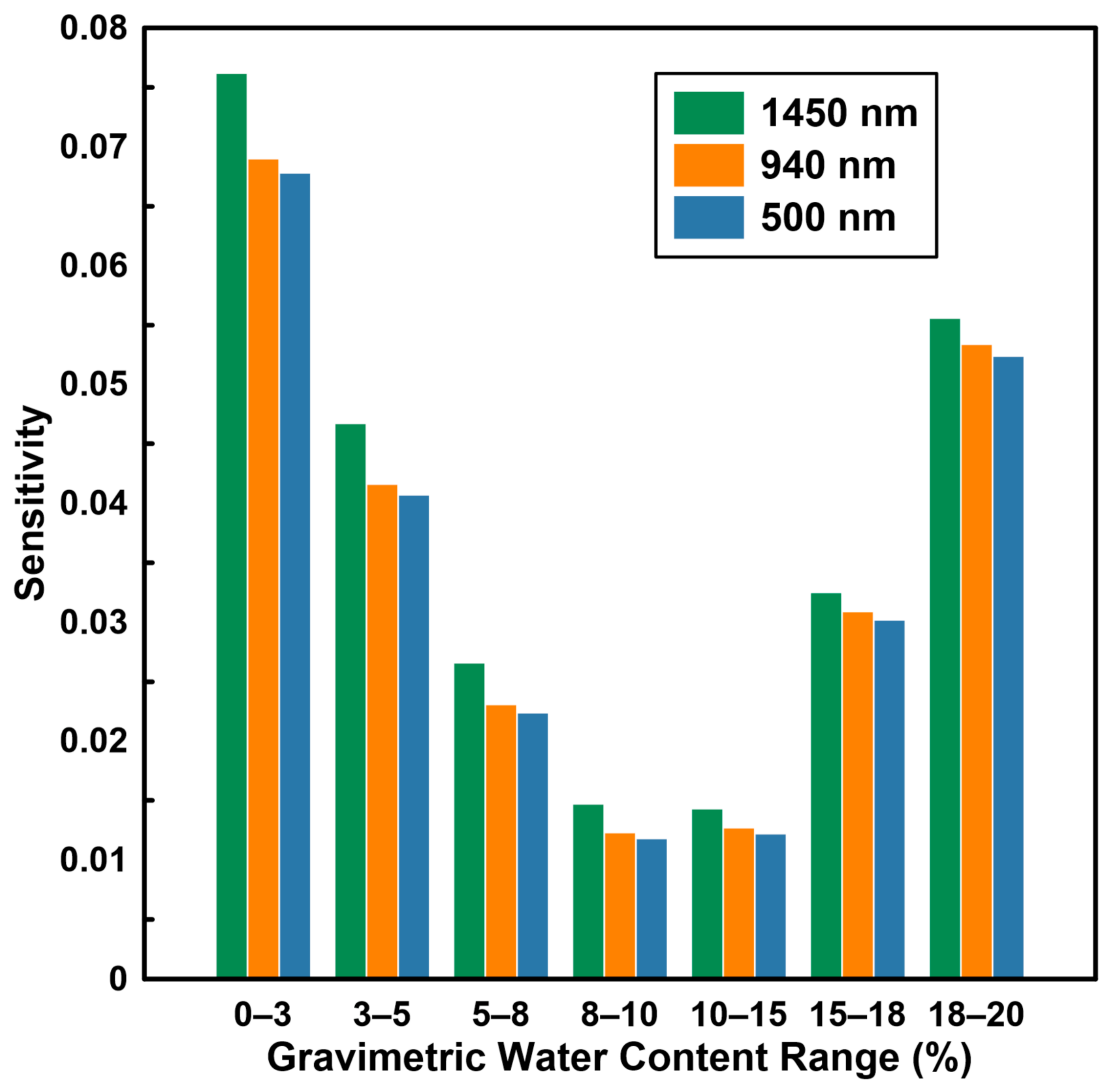
Shows that sensitivity varied across the tested water-content intervals and was generally highest at 1450 nm.

Because the fitted relationships were nonlinear, the sensor sensitivity varied across the investigated water-content range. Sensitivity was therefore calculated separately for each experimental interval using the change in predicted normalized photodiode output divided by the corresponding change in gravimetric water content. The results showed greater sensitivity under relatively dry and wet conditions and lower sensitivity within the intermediate water-content range. Among the evaluated wavelengths, 1450 nm generally provided the highest interval sensitivity, supporting its stronger moisture-dependent response. As shown in Figure 4, the sensor sensitivity varied across the water-content intervals, with the 1450 nm wavelength generally providing the highest range-dependent sensitivity.

**Figure 5:**
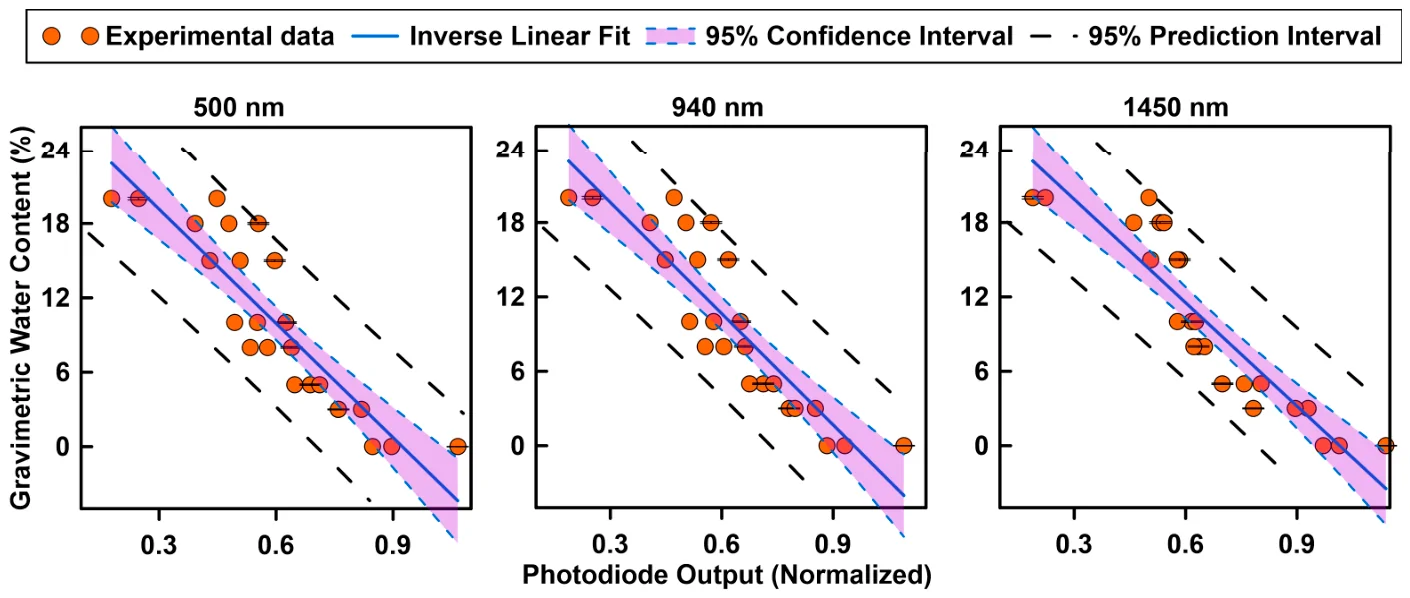
Inverse calibration of gravimetric water content using the combined-soil dataset at 500, 940, and 1450 nm. Solid lines show the linear inverse fits, shaded bands indicate 95% confidence intervals, and dashed lines represent 95% prediction intervals.

Direct inverse calibration was used to estimate GWC from the measured normalized photodiode output. The resulting combined-soil linear inverse models were:

(9) $$w=28.3598-30.7023\;I_{n,500}$$

(10) $$w=28.5917-29.9047\;I_{n,940}$$

(11) $$w=28.2100-27.7986\;I_{n,1450}$$

where $I_{n,500}$, $I_{n,940}$, and $I_{n,1450}$ are the normalized photodiode outputs at 500, 940, and 1450 nm, respectively. The inverse calibration relationships and their uncertainty bands are shown in Figure 5. Leave-one-water-content-level-out cross-validation produced RMSE values of 3.41, 3.37, and 3.20 percentage points of GWC at 500, 940, and 1450 nm, respectively, with corresponding MAE values of 2.85, 2.83, and 2.78 percentage points. The 1450 nm model therefore showed the lowest prediction error among the three wavelengths. These results represent internal validation and do not replace validation using independently prepared specimens.

After establishing the relationships between soil water content and the normalized photodiode output, the time-dependent behavior of the U-fiber sensor was evaluated. This analysis was conducted to examine the sensor response pattern, repeatability, and its ability to track moisture redistribution during the drying process.

**Figure 6:**
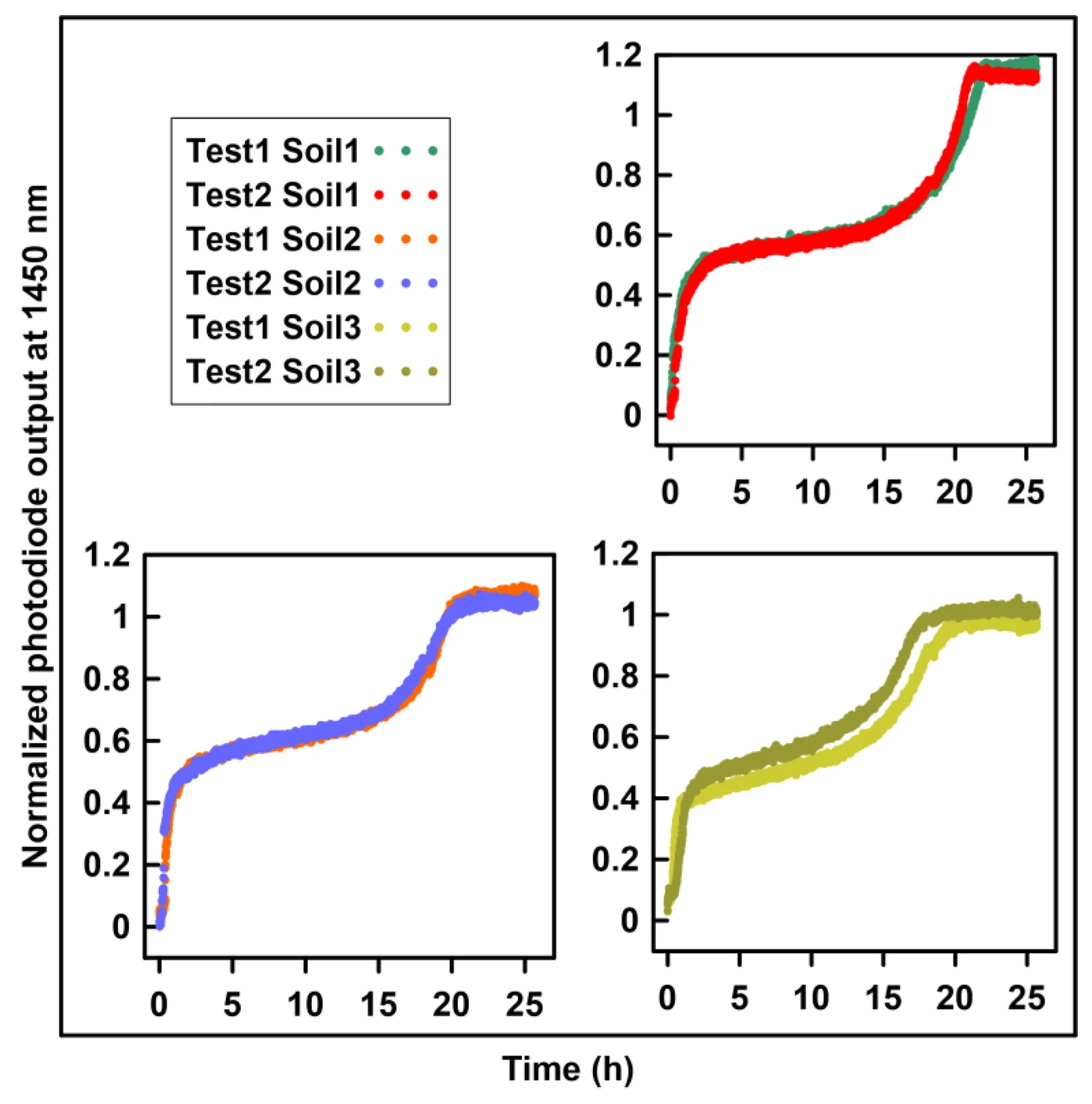
Temporal response of the U-shaped fiber optic sensor at 1450 nm during soil evaporation for repeated tests of the three soil samples.

During the evaporation test, the U-shaped fiber optic sensor was scanned using the 1450 nm wavelength to evaluate its ability to monitor temporal changes in soil moisture during drying. As shown in Figure 6, the normalized photodiode output increased with time for all tested soils, which is consistent with the relationship results because the optical output increases as the soil water content decreases during evaporation. At the beginning of the test, a rapid increase in signal was observed, followed by a slower transition region, indicating gradual moisture redistribution and evaporation from the soil sample. After approximately 18–21 h, the signal increased more sharply and then approached a relatively stable plateau, suggesting that the soil near the sensing region reached a lower moisture condition. The repeated tests showed similar overall trends, although small differences were observed between soils and repetitions. These variations can be attributed to differences in soil texture, color, packing condition, and soil–fiber contact. Overall, the 1450 nm response demonstrated that the developed U-shaped fiber optic sensor can track moisture variation during evaporation and can be used for continuous monitoring.

## 4. Discussion

The developed U-shaped fiber optic sensor exhibited a clear optical response to changes in gravimetric water content across the three tested soils and wavelengths. In general, the normalized photodiode output decreased as water content increased, indicating increased optical attenuation within the U-shaped sensing region. The measured response is likely governed by a combination of bending-induced optical loss, changes in the optical boundary condition surrounding the silica surface, water absorption, particle scattering, and soil–probe contact. Therefore, the observed signal variation should not be attributed exclusively to a single evanescent-wave mechanism.

The nonlinear response observed in Figure 3 is reasonable because the optical signal depends not only on water content but also on local pore-water distribution, particle arrangement, soil–probe contact, and scattering around the sensing region. These factors also explain the differences among the soil-specific calibration curves. The stronger descriptive fits obtained for individual soils compared with the combined-soil models indicate that soil characteristics influence the optical response and should be considered when developing generalized calibration relationships.

A clear wavelength-dependent response was observed at 500, 940, and 1450 nm. At 500 nm, particle color, surface reflectance, and scattering are expected to contribute substantially to the measured response. At 940 nm, both scattering and moisture-related near-infrared absorption may influence optical transmission. In contrast, 1450 nm lies within a strong water-absorption region and produced the strongest overall response to moisture variation. For the combined-soil dataset, the cubic model at 1450 nm provided the highest descriptive performance ($R^{2}=0.8956$, adjusted $R^{2}=0.8799$) and the lowest RMSE (0.0717) among the investigated wavelengths. These results support the use of 1450 nm as the most effective wavelength within the present sensing configuration.

The scatter observed in the combined-soil dataset is consistent with differences in particle-size range, visual color, packing condition, and soil–probe contact among the tested soils. Reinstallation of the probe can also introduce small changes in the local contact condition around the curved sensing region. The spring-supported platform was incorporated to reduce excessive loading and improve repeatability of contact; however, some installation-related variability remained. Only intra-sensor repeatability was evaluated, while sensor-to-sensor reproducibility was not assessed. These observations emphasize the importance of controlling specimen preparation and probe placement when applying this type of optical sensor.

The range-dependent sensitivity analysis further showed that the sensor response was nonlinear across the investigated water-content range. As shown in Figure 4, sensitivity was generally greater under relatively dry and wet conditions and lower at intermediate water contents. The 1450 nm wavelength showed the highest interval sensitivity across the evaluated ranges, further supporting its stronger moisture-dependent response.

For practical water-content estimation, the forward cubic relationships were separated from the inverse calibration models. Although the cubic models provided useful characterization of the nonlinear optical response, some soil-specific cubic relationships were not strictly monotonic and could therefore produce ambiguous water-content estimates if directly inverted. The direct inverse linear models avoided this problem by providing a unique water-content estimate from the measured normalized signal. As shown in Figure 5, grouped leave-one-water-content-level-out cross-validation produced RMSE values of 3.41, 3.37, and 3.20 percentage points of GWC at 500, 940, and 1450 nm, respectively, with 1450 nm again providing the lowest prediction error. These results represent internal predictive evaluation and should not be interpreted as independent external validation.

The evaporation experiments in Figure 6 provided an additional qualitative assessment of the temporal sensor response during drying. The normalized output generally increased as drying progressed, consistent with the fixed-water-content experiments. However, because intermediate gravimetric water content was not independently measured during the evaporation tests, these results should be interpreted as evidence of a repeatable temporal optical response rather than quantitative reconstruction of water content during drying. Controlled wetting experiments were not conducted; therefore, wetting–drying hysteresis in the sensor response was not evaluated.

Overall, the results demonstrate the feasibility of the U-shaped silica optical sensing approach for soil water-content monitoring under controlled laboratory conditions. The optical configuration also avoids some limitations associated with electrical sensing methods, such as direct dependence on electrode conductivity and electrical contact. Nevertheless, further work is required before field application. Response and recovery times were not experimentally quantified in the present study and should be systematically evaluated in future work. Future studies should include independently prepared replicate specimens, additional soil types, controlled variations in dry density, salinity, and temperature, and external validation of the inverse calibration models. Controlled wetting and drying cycles should also be conducted to evaluate potential wetting–drying hysteresis in the sensor response. Improved mechanical control of probe installation and direct comparison with established soil water-content sensors would also help determine the practical measurement performance of the proposed system.

## 5. Conclusions

This study developed and evaluated a U-shaped silica fiber-optic sensor for gravimetric soil water-content measurement using normalized photodiode responses at 500, 940, and 1450 nm. The sensing platform combined a U-shaped silica probe with LED light sources, a concave-mirror coupling unit, and photodiode detection, providing a relatively simple optical configuration without active heating, fiber Bragg grating interrogation, or spectrometer-based measurement.

The normalized optical response generally decreased as gravimetric water content increased for all three tested soils. Among the evaluated forward models, the third-order polynomial provided the strongest descriptive representation of the nonlinear sensor response. For the combined-soil dataset, the 1450 nm model showed the best overall descriptive performance, with $R^{2}=0.8956$, adjusted $R^{2}=0.8799$, and RMSE = 0.0717. The stronger response at 1450 nm is consistent with its location within a strong water-absorption region.

The soil-specific models generally showed stronger descriptive fits than the combined-soil models, indicating that particle-size range, soil color, packing condition, and soil–probe contact influenced the optical response. The sensitivity analysis also showed a nonlinear, range-dependent response, with 1450 nm providing the highest sensitivity across the evaluated water-content intervals.

For practical estimation of unknown water content, separate direct inverse linear calibration models were developed. Leave-one-water-content-level-out cross-validation produced RMSE values of 3.41, 3.37, and 3.20 percentage points of GWC at 500, 940, and 1450 nm, respectively, with corresponding MAE values of 2.85, 2.83, and 2.78 percentage points. The 1450 nm inverse model therefore provided the lowest internal prediction error among the three wavelengths. These results represent internal validation and should not be interpreted as independent external validation.

The evaporation experiments further demonstrated a repeatable qualitative optical response during progressive drying. Because intermediate gravimetric water content was not independently measured during these tests, the temporal results should be interpreted as qualitative rather than as a quantitative reconstruction of water content.

Overall, the proposed U-shaped silica fiber-optic sensor demonstrated clear sensitivity to soil water-content variation under controlled laboratory conditions. Further work should include independently prepared replicate specimens, additional soil types, controlled variations in dry density, salinity, and temperature, improved control of probe–soil contact, and external validation before field application.

## Figures and Tables

**Table 1:** Comparison of representative soil-moisture measurement technologies.

Method	Principle	Main Advantages	Main Limitations	References
Gravimetric method	Mass loss after oven drying	Direct and accurate reference method	Destructive, labor-intensive, and unsuitable for continuous monitoring	[5]
TDR	Electromagnetic travel time related to dielectric permittivity	Accurate, rapid, and suitable for continuous monitoring	Relatively expensive; affected by salinity and soil-specific conditions	[6,22]
FDR/capacitance	Frequency or capacitance variation caused by changes in dielectric permittivity	Low cost, simple operation, and easy integration	Sensitive to salinity, temperature, density, and soil-specific calibration	[7,22,23,24]
Neutron probe	Neutron moderation by hydrogen atoms in soil	Large sensing volume and suitable for depth profiling	Radiation-safety requirements, high cost, and limited near-surface resolution	[8]
Actively heated fiber optics	Soil thermal response around a heated optical fiber	Distributed measurement and electromagnetic immunity	Requires heating power, calibration, and optical interrogation equipment	[11,13]
Fiber Bragg grating sensor	Moisture-induced wavelength or thermal-response shift	Small size, multiplexing capability, and high sensitivity	Temperature cross-sensitivity and relatively complex interrogation	[12,25]
Coated or modified optical fiber	Moisture-dependent resonance, refractive-index, or transmission variation	High sensitivity and electromagnetic immunity	Requires fiber modification, functional coatings, and specialized fabrication	[17,18,19,20,21]

**Table 2:** Specifications of the LED light sources used in the sensor.

LED	Manufacturer	Model Number	Peak Wavelength (nm)	Spectral FWHM	Rated Emission Output
Visible LED	Bivar, Irvine, CA, USA	3UTC-F	500	≈20 nm at 20 mA	800 mcd at 20 mA *
Near-infrared LED	Vishay Semiconductors, Mumbai, India	TSAL6400	940	30 nm at 100 mA	40 mW at 100 mA
Near-infrared LED	Marktech Optoelectronics, Latham, NY, USA	MTE6014-526-IR	1450	150 nm at 20 mA	3.6 mW at 20 mA; 13 mW at 100 mA

* mcd = Millicandela, a unit of luminous intensity equal to $10^{-3}$ candela (cd).

**Table 3:** Soil samples and water content levels used to evaluate the sensor response.

Soil Sample	Soil Type	Visual Color	Point	Repetition	Water Content (%)
Soil 1			1	5	0, 3, 5, 8, 10, 15, 18, and 20%
	Sand 30/40	White	2	5	
			3	5	
Soil 2			1	5	0, 3, 5, 8, 10, 15, 18, and 20%
	Sand 40/50	Dark brown	2	5	
			3	5	
Soil 3			1	5	0, 3, 5, 8, 10, 15, 18, and 20%
	Sand 100/200	Light brown	2	5	
			3	5	

## Data Availability

The data supporting the findings of this study are available from the corresponding author upon reasonable request.

## Supplementary Materials

The following supporting information can be downloaded at: https://www.mdpi.com/article/10.3390/s26175619/s1, Table S1: Comparison of candidate forward response models for the combined-soil dataset. Table S2: Combined-soil cubic regression models based on 24 condition-level observations. Table S3: Soil-specific cubic regression coefficients and goodness-of-fit statistics based on eight condition-level values per soil.
