# Supplementary material for: Development and Performance Analysis of a U-Shaped Fiber Optic Sensor for Soil Water Content Measurement
Source: Sensors (Basel). 2026 Sep 4;26(17):5619. doi: 10.3390/s26175619 (PMC13567911; doi:10.3390/s26175619)
Supplement: Supplementary file 1 [file sensors-26-05619-s001.zip › sensors-4469153-supplementary.pdf]

# Supplementary Materials for: Development and Performance Analysis of a U-Shaped Fiber Optic Sensor for Soil Water Content Measurement

**Table S1**. Comparison of candidate forward response models for the combined-soil dataset.

| Wavelength | Model | $R^2$ | Adjusted $R^2$ | RMSE | AICc |
|---|---|---|---|---|---|
| 500 nm | Linear | 0.7979 | 0.7887 | 0.0886 | −111.77 |
| 500 nm | Quadratic | 0.8168 | 0.7994 | 0.0843 | −111.51 |
| 500 nm | Cubic | 0.8631 | 0.8426 | 0.0729 | −115.59 |
| 500 nm | Exponential | 0.8197 | 0.8115 | 0.0837 | −114.52 |
| 940 nm | Linear | 0.8014 | 0.7924 | 0.0903 | −110.83 |
| 940 nm | Quadratic | 0.8189 | 0.8017 | 0.0863 | −110.41 |
| 940 nm | Cubic | 0.8649 | 0.8446 | 0.0745 | −114.53 |
| 940 nm | Exponential | 0.8220 | 0.8139 | 0.0855 | −113.45 |
| 1450 nm | Linear | 0.8305 | 0.8228 | 0.0914 | −110.27 |
| 1450 nm | Quadratic | 0.8501 | 0.8359 | 0.0859 | −110.60 |
| 1450 nm | Cubic | 0.8956 | 0.8799 | 0.0717 | −116.36 |
| 1450 nm | Exponential | 0.8537 | 0.8470 | 0.0849 | −113.80 |

**Table S2**. Combined-soil cubic regression models based on 24 condition-level observations.

| Wavelength | $Y = a_0 + a_1w + a_2w^2 + a_3w^3$ | $R^2$ | Adjusted $R^2$ | RSS | RMSE | AICc |
|---|---|---|---|---|---|---|
| 500 nm | $Y = 0.953794 - 0.087348w + 0.007169w^2 - 0.000219w^3$ | 0.8631 | 0.8426 | 0.1275 | 0.0729 | −115.59 |
| 940 nm | $Y = 0.986383 - 0.089139w + 0.007322w^2 - 0.000225w^3$ | 0.8649 | 0.8446 | 0.1333 | 0.0745 | −114.53 |
| 1450 nm | $Y = 1.062130 - 0.098856w + 0.008028w^2 - 0.000245w^3$ | 0.8956 | 0.8799 | 0.1235 | 0.0717 | −116.36 |

**Table S3**. Soil-specific cubic regression coefficients and goodness-of-fit statistics based on eight condition-level values per soil.

| Soil | Wavelength | $a_0$ | $a_1$ | $a_2$ | $a_3$ | $R^2$ | RMSE | RSS |
|---|---|---|---|---|---|---|---|---|
| Soil 1 | 500 nm | 1.069626 | −0.141081 | 0.014587 | −0.000475 | 0.9779 | 0.0316 | 0.00799 |
| Soil 1 | 940 nm | 1.094179 | −0.142499 | 0.014848 | −0.000487 | 0.9777 | 0.0325 | 0.00845 |
| Soil 1 | 1450 nm | 1.143501 | −0.163272 | 0.016649 | −0.000539 | 0.9805 | 0.0346 | 0.00959 |
| Soil 2 | 500 nm | 0.916005 | −0.050620 | 0.001703 | −0.000015 | 0.9789 | 0.0225 | 0.00407 |
| Soil 2 | 940 nm | 0.951667 | −0.052556 | 0.001856 | −0.000020 | 0.9785 | 0.0233 | 0.00434 |
| Soil 2 | 1450 nm | 1.036717 | −0.058548 | 0.002154 | −0.000026 | 0.9759 | 0.0274 | 0.00600 |
| Soil 3 | 500 nm | 0.875751 | −0.070343 | 0.005218 | −0.000167 | 0.9637 | 0.0382 | 0.01168 |
| Soil 3 | 940 nm | 0.913302 | −0.072362 | 0.005262 | −0.000167 | 0.9630 | 0.0403 | 0.01297 |
| Soil 3 | 1450 nm | 1.006170 | −0.074748 | 0.005280 | −0.000169 | 0.9579 | 0.0468 | 0.01749 |
